# Late Simultaneous Metastasis of Renal Cell Carcinoma to the Submandibular and Thyroid Glands Seven Years after Radical Nephrectomy

**DOI:** 10.1155/2010/698014

**Published:** 2010-07-25

**Authors:** Mohammed S. Miah, Sharon J. White, George Oommen, Esther Birney, Samit Majumdar

**Affiliations:** ^1^Department of Otolaryngology-Head & Neck Surgery, Ninewells Hospital & Medical School, Dundee DD1 9SY, UK; ^2^Department of Pathology, Ninewells Hospital & Medical School, Dundee DD1 9SY, UK

## Abstract

*Background*. Renal cell carcinoma (RCC) metastasis to the salivary glands is extremely rare. Most cases reported previously have involved the parotid gland and only six cases involving the submandibular gland exist in the current literature. Metastasis of RCC to thyroid gland is also rare but appears to be more common than to salivary glands. *Methods and Results*. We present the first case of simultaneous metastasis to the submandibular and thyroid glands from clear cell RCC in a 61-year-old woman who presented seven years after the primary treatment. The submandibular and thyroid glands were excised completely with preservation of the marginal mandibular and recurrent laryngeal nerves, respectively. *Conclusion*. Metastatic disease should always be considered in the differential diagnosis for patients who present with painless salivary or thyroid gland swelling with a previous history of RCC. If metastatic disease is confined only to these glands, prompt surgical excision can be curative.

## 1. Introduction

Metastatic salivary and thyroid gland tumours are very rare among reported clinical cases. With regards to salivary gland metastasis, often the primary site of origin of tumour is the skin or mucosal lining of the head and neck structures, with melanomas and squamous cell carcinomas being the most common primary tumours, respectively [1]. In metastatic thyroid tumours, the most common sites of primary tumours are the breast, kidney, and lung, and in some cases they are detected only at autopsy [2]. RCC is an unpredictable tumour that can recur many years after the original diagnosis and metastasis of this tumour to the salivary and thyroid glands is an uncommon occurrence that can cause clinical and pathological problems in diagnosis [3].

In this paper we describe a patient in whom simultaneous metastatic submandibular and thyroid gland tumours occurred seven years after primary radical nephrectomy for RCC.

## 2. Case Report

A 61-year-old Caucasian female presented to her general practitioner with painless and palpable right-sided submandibular and thyroid swellings. Her past history revealed left mastectomy and axillary clearance with postoperative chemoradiotherapy for grade 2 lobular breast carcinoma 2 years ago, and left radical nephrectomy for clear cell type RCC (Fuhrman grade 2, stage pT1, completely excised with no renal vein involvement) with no regional or distant metastases 7 years ago. Considering her recent history of invasive breast carcinoma, she was referred onto the breast surgical clinic for further assessment. An ultrasound scan (US) of her neck suggested multinodular thyroid goitre (largest nodule in right lobe) and a well-defined hypoechoic mass in the right submandibular gland. US-guided fine-needle aspiration (FNA) cytology of the right thyroid nodule was nondiagnostic on two separate occasions and of the right submandibular gland suggested a pleomorphic adenoma (PA). 

**Figure 1:**
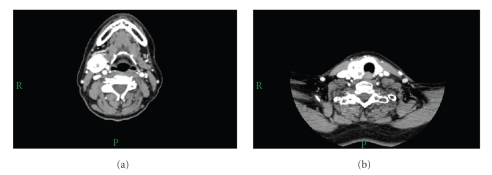
CT of neck with contrast. (a) Well-defined tumour mass in the right submandibular gland measuring 29 × 26 × 30 mm. It shows prominent vascularisation which would be unusual for benign lesion such as PA. There is some low-density area centrally in the tumour probably related to necrosis. (b) There is a large nodule in the thyroid on the right side measuring 26 × 23 × 30 mm showing similar intense enhancement as the submandibular lesion.

She was then seen in our otolaryngology department where a complete ear, nose, and throat examination was normal except for the obvious right submandibular and thyroid gland swellings. A computed tomography (CT) scan of her neck was performed which revealed a tumour mass in the right submandibular gland with prominent vascularisation unusual for benign lesions such as a PA (Figure 1). CT scan also showed a large nodule in the right thyroid with similar intense vascular enhancement as the submandibular lesion (Figure 1). There was no suspicious cervical lymphadenopathy. A bone scan showed no osseous metastases and chest X-ray revealed no pulmonary metastases.

**Figure 2:**
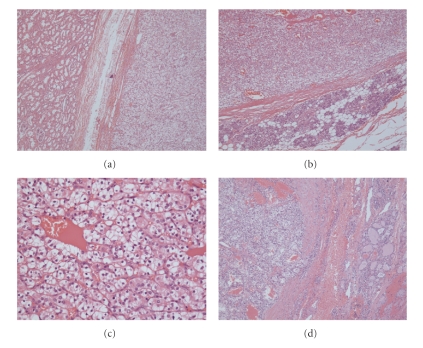
(a) Original renal tumour (H&E stain, original magnification x40). On the left is normal kidney and clear cell RCC is on the right. (b) Submandibular gland (H&E stain, original magnification x40): metastatic RCC on the top with normal glandular tissue in the lower portion. (c) Metastatic RCC in submandibular gland (H&E stain, original magnification x200). (d) Thyroid tumour (H&E stain, original magnification x40): tumour on the left and normal thyroid tissue on the right.

Initially the right submandibular gland was excised revealing an encapsulated tumour within the glandular tissue. A right level I and II neck dissection was also performed with preservation of the marginal mandibular nerve. Histopathological examination of the tumour showed striking similarity to the clear cell RCC of left kidney resected previously (Figure 2). The diagnosis was further supported by immunohistochemical findings that showed positive staining for vimentin, CD10, low molecular weight cytokeratins, epithelial membrane, and RCC antigens, and negative for carcinoembryonic antigen (CEA), S100, and CD34. Subsequently the patient's case was discussed at the combined surgical oncology and pathology meeting, and due to the suspicious nature of the thyroid gland, a total thyroidectomy was performed with preservation of the recurrent laryngeal nerves and parathyroid glands. Histopathological analysis of right lobe of thyroid gland confirmed metastatic clear cell RCC similar to the submandibular gland disease (Figure 2). As there was no extracapsular spread and no lymphatic disease, no further adjuvant therapy was given to the patient. At 6-month postoperative followup, the patient showed no signs of recurrence or other metastases.

## 3. Discussion

In adults, RCC constitutes approximately 2%-3% of all malignancies representing 85% of all primary renal tumours, and its incidence increases with age, with a peak in the sixth decade of life [4, 5]. These tumours constitute a group of epithelial tumours that are highly heterogeneous, consisting of several histological subtypes demonstrating different biological profiles and clinical behaviour [5, 6]. Clear cell RCC is the most commonly occurring, predominantly associated with mutations involving the von Hippel-Lindau gene and elaboration of vascular and somatic factors; thus these tumours typically exhibit hypervascularity at imaging [6]. These cancer cells are also known to have a good adaptive potential in a diverse array of microenvironments, thus giving rise to a high metastatic potential of RCC [1]. After nephrectomy for earlier stages of RCC, up to 50% of patients develop recurrent or metastatic disease, with 85% of recurrences occurring within 3 years after primary resection but being reported up to several decades later [7]. 

The most frequent sites for RCC metastasis are the lung, bone, liver, adrenal gland, contralateral kidney, retroperitoneum, brain, and skin [1, 7]. Metastatic RCC to the head and neck region is less common, comprising only 8%–14% of all cases, with thyroid gland being the most common site [1]. Although uncommon, more than 150 cases of clinically recognized metastatic renal cell carcinoma to the thyroid have been reported in the English literature. Most cases of metastatic thyroid tumours originate from primary renal tumours [8]. Metastatic disease from the kidney to the thyroid gland can occur more than 20 years after nephrectomy with the average time interval being 7.5 years [9]. 

**Figure 3:**
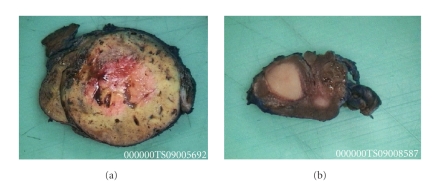
Macroscopic specimens showing metastatic RCC within the submandibular (a) and thyroid (b) glands. Solid metastatic tumours can be seen to be surrounded by normal glandular tissue in both cases without any extraglandular extension.

Metastasis of RCC to the major salivary glands is extremely rare. Our search only found a handful of reported cases with a predominance of parotid gland metastases. Based on our search, only six cases of RCC metastasis to the submandibular gland exist in the current literature (one of the six reports describes RCC metastasis to Wharton's duct) [10]. Our case is unique with simultaneous solitary metastases within the submandibular and thyroid glands, surrounded by normal glandular tissue, with no evidence of extraglandular extension (Figure 3). We cannot speculate a pathogenetic mechanism to explain this phenomenon at this stage.

Radiologically it is difficult to distinguish between metastatic and primary malignant neoplasms [1, 7]. In our case, US scan and FNA were suggestive of benign disease in both sites (multinodular goitre and PA). It is the clinical suspicion of malignancy leading to CT scanning of the patient's neck that raised further suspicion of a malignant process, thus the surgical excision of the submandibular and thyroid glands. One of the most important issues is to differentiate metastatic RCC from other submandibular gland neoplasms and primary clear cell thyroid neoplasms. The use of periodic-acid Shiff (PAS) with enzymes, such as diastase and mucin stains, is helpful in distinguishing primary submandibular gland tumours which may have clear cell areas, such as acinic cell and mucoepidermoid carcinomas from RCC [1]. Generally, RCC contains glycogen, making it PAS positive and diastase negative; on the other hand, acinic cell carcinoma and mucoepidermoid carcinoma are revealed as PAS positive and contain diastase-resistant material [1]. Mucin stains will aid in the diagnosis of mucoepidermoid carcinoma. In the case of clear cell thyroid gland tumours, the use of thyroglobulin immunohistochemistry can help to distinguish from metastatic RCC [11]. 

In addition, RCC shows immunohistochemically positive staining with low molecular weight cytokeratins, CD10, vimentin, and RCC antigen, and CEA negativity [12]. In our case, the neoplastic cells showed positive immunostaining for vimentin, low molecular weight cytokeratins, CD10, and RCC antigen but were immunonegative for CEA, thus confirming the diagnosis of metastatic RCC.

## 4. Conclusion

Metastatic disease should always be considered in the differential diagnosis for patients who present with painless salivary or thyroid gland swelling with a previous history of renal cell carcinoma. Ultrasound and cytological findings may not always reflect the actual pathology and therefore further imaging is paramount in such circumstances. If metastatic disease is confined to these glands only, prompt surgical excision can be curative.
